# Layers of dendritic cell-mediated T cell tolerance, their regulation and the prevention of autoimmunity

**DOI:** 10.3389/fimmu.2012.00183

**Published:** 2012-07-03

**Authors:** Christian T. Mayer, Luciana Berod, Tim Sparwasser

**Affiliations:** Institute of Infection Immunology, TWINCORE, Centre for Experimental and Clinical Infection Research; a joint venture between the Medical School Hannover (MHH) and the Helmholtz Centre for Infection Research (HZI)Hannover, Germany

**Keywords:** DC, tolerance, Foxp3, Treg, CD103, autoimmunity, infection

## Abstract

The last decades of Nobel prize-honored research have unequivocally proven a key role of dendritic cells (DCs) at controlling both T cell immunity and tolerance. A tight balance between these opposing DC functions ensures immune homeostasis and host integrity. Its perturbation could explain pathological conditions such as the attack of self tissues, chronic infections, and tumor immune evasion. While recent insights into the complex DC network help to understand the contribution of individual DC subsets to immunity, the tolerogenic functions of DCs only begin to emerge. As these consist of many different layers, the definition of a “tolerogenic DC” is subjected to variation. Moreover, the implication of DCs and DC subsets in the suppression of autoimmunity are incompletely resolved. In this review, we point out conceptual controversies and dissect the various layers of DC-mediated T cell tolerance. These layers include central tolerance, Foxp3^+^ regulatory T cells (Tregs), anergy/deletion and negative feedback regulation. The mode and kinetics of antigen presentation is highlighted as an additional factor shaping tolerance. Special emphasis is given to the interaction between layers of tolerance as well as their differential regulation during inflammation. Furthermore, potential technical caveats of DC depletion models are considered. Finally, we summarize our current understanding of DC-mediated tolerance and its role for the suppression of autoimmunity. Understanding the mechanisms of DC-mediated tolerance and their complex interplay is fundamental for the development of selective therapeutic strategies, e.g., for the modulation of autoimmune responses or for the immunotherapy of cancer.

## Introduction

Tolerance can be defined by the complete absence or partial inhibition of a potentially harmful adaptive immune response. Immunological tolerance operates continuously in order to protect mammals not only from the deleterious attack of self tissues, but also from the rejection of semi-allogeneic offspring and from uncontrolled immune responses against foreign antigens (Goodnow et al., 2005; Trowsdale and Betz, 2006; Erlebacher, 2010; Berod et al., 2012). Therefore, although tolerance is a prerequisite for the existence of mammal species, excessive tolerance can become similarly life threatening, e.g., by circumventing cancer immunosurveillance, or by dampening pathogen-specific immunity and thereby causing lethal or chronic infections (Zou, 2006; Belkaid, 2007; Berod et al., 2012). Thus, a fine-tuned balance between tolerance and immunity allows for the specific attack and clearance of dangerous pathogens and transformed cells, whereas harmless self tissues, commensal microorganisms, food- and environmental antigens remain ignored. The risk of tolerance dysregulation and autoimmunity may, however, be the evolutionary price of having a highly specialized immune system. Indeed, a recent study identified pathogens as the major driving force for the local genetic adaptation of humans, suggesting co-evolution between microbes and the human immune system (Fumagalli et al., 2011). Strikingly, gene loci that confer susceptibility for autoimmune diseases including type I diabetes, colitis, and rheumatoid arthritis, represent prominent targets for this pathogen-driven selection (Fumagalli et al., 2011). Thus, although such alleles may have evolved for better pathogen control, they likely also contribute to autoimmunity, exemplifying that tolerance and immunity are two sides of a double-edged sword.

Dendritic cells (DCs) populate virtually all tissues and serve as sentinels equipped to detect potential threats. Rapidly, they induce responses in order to protect the host. These include innate immune cell activation and recruitment, inflammation, orchestration of adaptive immunity, and immunological memory. Thus, DCs are “natural adjuvants” bridging innate and adaptive immunity and have clinically been exploited for almost a century to prevent life-threatening infections by vaccination. Additionally, by integrating environmental signals, DCs critically balance tolerance and immunity (Banchereau and Steinman, 1998; Hawiger et al., 2001; Probst et al., 2005).

DC-mediated tolerance can be segregated into at least four layers: central tolerance, Foxp3^+^ regulatory T cells (Tregs), anergy/deletion and feedback regulation. Additionally, interactions between these layers need to be considered. Although insufficient insights into the regulation of individual layers is still a limiting factor in resolving the complexity of DC-mediated tolerance, central aims of this review are (1) to dissect the multiple layers of DC-mediated tolerance according to current knowledge; (2) to discuss whether tolerance mechanisms are inherent to or acquired by particular DC subsets and how individual layers of tolerance are regulated by inflammation; and (3) to examine the role of DCs for the suppression of autoimmunity.

## DCs and Foxp3^+^ tregs: guardians of T cell immunity

DCs are heterogeneous with respect to subset composition, localization and function. The DC network across the tissues of different mammal species was recently classified into five conserved subsets. These subsets share common developmental requirements, gene expression patterns, and/or functional specializations and comprise: two types of conventional DCs (CD8α-like cDCs and CD11b-like cDCs), plasmacytoid DCs (pDCs), monocyte-derived DCs (Mo-DCs), and Langerhans cells (LCs) (reviewed in Geissmann et al., 2010; Guilliams et al., 2010a,b; Bar-on et al., 2011b; Belz and Nutt, 2012). Although this simplified model helps to understand parallels between mice and men, markers to identify DC subsets differ substantially between species. In mice, CD8α-like cDCs comprise lymphoid organ-resident CD8α^+^CD103^+/−^CD11b^−^DEC-205^+^DNGR-1^+^ cDCs and non-lymphoid tissue migratory CD8α^−^CD103^+^CD11b^−^DEC-205^+^DNGR-1^−^ cDCs (Sancho et al., 2008; Ginhoux et al., 2009; Del Rio et al., 2010; Edelson et al., 2010). Both subsets will be summarized as CD8α/CD103^+^ cDCs in the following. CD11b-like cDCs (later called CD11b^+^ cDCs) include lymphoid organ-resident and non-lymphoid tissue migratory CD11b^+^ cDCs, both being heterogeneous and comprising further subgroups (Ginhoux et al., 2009; Guilliams et al., 2010b; Henri et al., 2010). While CD11b^+^ cDCs are more specialized in innate responses and CD4^+^ T cell immunity, CD8α/CD103^+^ cDCs are efficient at cross-presenting exogenous antigens (Guilliams et al., 2010b). The intestinal lamina propria is the only known site populated by CD11b^+^ Mo-DCs in the steady state and it further contains a CD103^+^CD11b^+^ migratory cDC subset with unique developmental and functional characteristics (Jaensson et al., 2008; Bogunovic et al., 2009; Ginhoux et al., 2009; Schulz et al., 2009; Helft et al., 2010).

One critical component of DC-mediated tolerance is the interaction of DCs with immunosuppressive CD4^+^ Tregs (Tarbell et al., 2004). Foxp3 was identified as a master regulator of CD4^+^ Treg function through transcriptional control of around 1100 target genes (Fontenot et al., 2003; Hori et al., 2003; Khattri et al., 2003; Apostolou et al., 2008). However, Foxp3 itself is dispensable for Treg lineage commitment and thus for part of the Treg-specific transcriptome (Lin et al., 2007; Lahl et al., 2009). Foxp3 is highly restricted to CD4^+^TCRαβ^+^ Tregs in mice with the exception of its transient expression by a minor population of non-Tregs (Komatsu and Hori, 2007; Liston et al., 2007; Sakaguchi et al., 2008; Kim et al., 2009; Wolf et al., 2010; Mayer et al., 2012; Miyao et al., 2012). Moreover, Foxp3 is expressed by few CD8^+^ T cells that were recently shown to expand after allogeneic bone marrow transplantation (Mayer et al., 2011; Robb et al., 2012). Of note, in humans additional markers like CD45RA and CD45RO help to discriminate Foxp3^+^ Tregs from activated T cells (Sakaguchi et al., 2010).

Foxp3^+^ Tregs comprise two major subsets, natural and induced Tregs. Both subsets are devoted to immune suppression and most likely act in a complementary fashion by extending T cell receptor diversity (Haribhai et al., 2011). Natural Foxp3^+^ Tregs develop as distinct T cell lineage in the thymus in a DC-independent fashion (Ohnmacht et al., 2009). In contrast, DCs critically control induced Foxp3^+^ Treg generation from Foxp3^−^ T cells in the periphery. Additionally, DCs tailor the peripheral homeostasis of both natural and induced Foxp3^+^ Tregs (see “DCs and Foxp3^+^ Tregs”). Requirements for natural and induced Foxp3^+^ Treg development, as well as their immunosuppressive activities, have previously been reviewed (Curotto De Lafaille and Lafaille, 2009; Huehn et al., 2009; Josefowicz and Rudensky, 2009; Sakaguchi et al., 2009; Ohkura and Sakaguchi, 2010; Berod et al., 2012; Hsieh et al., 2012). However, these issues are far from being completely solved.

## Tolerogenic DCs

The term “tolerogenic” is increasingly being used to characterize certain DC functions or DC subsets. However, its definition can be quite intuitive. For example, tolerogenicity has been attributed to DCs solely based on the expression of one or more immunosuppressive molecules. Conversely, reduced expression of immunostimulatory molecules has similarly been regarded as a tolerogenic property. Other definitions include the capacity of a DC to generate Tregs or its inability to generate a T cell response. Whether these T cell responses are measured *in vitro* or *in vivo* is up to the scientist's definition. Moreover, the use of *in vitro* generated or *ex vivo* isolated DCs adds an additional level of complexity. Finally, various protocols have been developed to generate “tolerogenic” DCs by pharmacologic treatment (reviewed in Maldonado and Von Andrian, 2010). These examples illustrate that the term “tolerogenic DC” is vaguely defined and requires refinement.

**Figure 1:**
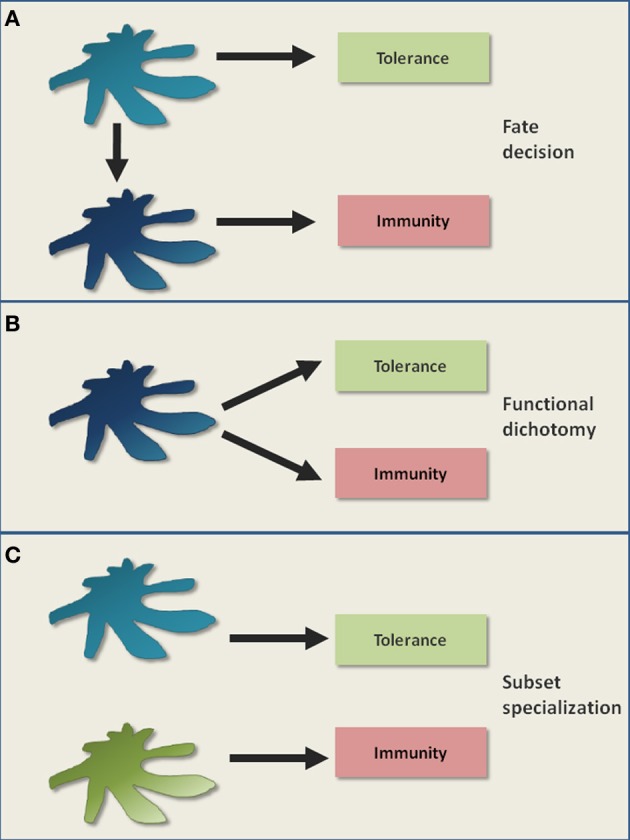
**Classical models of DC-mediated tolerance.** It is a long standing controversy how DCs mediate immunological tolerance. **(A)** One classical view is that the maturation status of DCs acts as a switch, determining the decision for either tolerance in the steady state (immature/semimature DCs) or for immunity upon inflammation (mature DCs). **(B)** The subsequent discovery of immunosuppressive regulatory T cells that can be activated by both immature and mature DCs, raised the possibility for functional dichotomy. This means that a DC is capable of potentially executing both tolerogenic and immunogenic immune responses as parallel events. **(C)** Certain DC subsets were proposed to be inherently more potent in tolerance induction compared to other DC subsets. In this model, a division of labor between DC subsets regulates tolerance versus immunity.

Figure 1 outlines different models of DC-mediated tolerance. Classically, DCs are believed to retain an immature/semi-mature steady state in order to primarily induce tolerance (Steinman et al., 2003). The triggering of diverse pattern recognition receptors, cytokine receptors or co-stimulatory receptors (e.g., through infections) lead to DC maturation (Figure 1A). Maturation is characterized by increased densities of MHC: peptide complexes and co-stimulatory molecules like CD80/CD86 on the surface of a DC, as well as the release of inflammatory cytokines and chemokines (Steinman et al., 2003). Matured DCs are considered to be potent stimulators of immunity (Figure 1A). However, as the mere expression of known co-stimulatory molecules like CD80/CD86 is not indicative of an immunogenic function, this classical view has been refined (Reis E Sousa, 2006). In this new model, maturation is rather seen functionally and results in the generation of immunogenic DCs, e.g., involving the licensing of DCs by CD4^+^ T cell help (Reis E Sousa, 2006). Thus, DC-mediated tolerance can be classically described by a situational fate decision where the DC either achieves functional maturation and becomes immunogenic, or fails to mature and promotes tolerance (Figure 1A).

A key question remains how matured DCs can distinguish between pathogen-specific T cells and T cells autoreactive to ubiquitous self antigens. In fact, the maintenance of self tolerance may become even more important during inflammatory conditions than during the steady state in order to prevent autoimmunity. Taking this into consideration, one can postulate that even matured immunogenic DCs continue to promote tolerance. In this model, DCs possess a functional dichotomy by promoting tolerance and immunity as parallel events (Figure 1B), although it fails to explain the abrogation of tolerogenic DC functions upon inflammation, as observed in certain experimental settings (Hawiger et al., 2001; Laffont et al., 2010).

**Figure 2:**
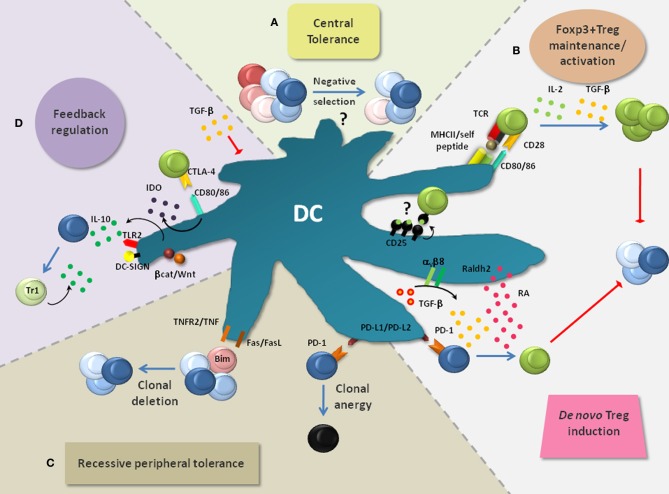
**Layers of DC-mediated tolerance.** DCs promote tolerance via multiple layers, thereby rendering the term “tolerogenic DC” highly indefinite. **(A)** DCs are implicated in the negative selection of self-reactive T cells and thus in central tolerance, although this is still a subject of intense research. **(B)** DCs are critically involved in the *de novo* generation, homeostasis and activation of Foxp3^+^ Tregs which play a non-redundant role in the suppression of lethal autoimmunity. Foxp3^+^ Treg activation by DC-associated MHC-class-II: peptide complexes and CD80/CD86 is crucial to activate the immunosuppressive properties of Foxp3^+^ Tregs and to induce their expansion. Additionally, IL-2 and TGF-β, which can be produced by DCs, are critically involved in the maintenance of Treg function and proliferation. DC-derived TGF-β can also act on Foxp3^−^ T cells to convert them into Foxp3^+^ Tregs. This process is enhanced by retinoic acid (RA) which is generated in DCs from retinaldehyde by the enzyme Raldh2. PD-1 ligands (PD-L1 and PD-L2) were also implied in Foxp3^+^ Treg induction. **(C)** Recessive peripheral tolerance is established in a T cell-intrinsic manner following instructive encounters with DCs. Antigenic activation of Foxp3^−^ T cells can result in their functional inactivation (clonal anergy). This partly depends on the triggering of PD-1 on T cells via its ligands PD-L1 and PD-L2. Similarly, signaling via CTLA-4 can induce clonal anergy (not depicted). Another outcome of recessive tolerance can be clonal T cell deletion which depends on interactions via TNF/TNFR2, FasL/Fas or induction of the pro-apoptotic factor Bim. **(D)** Multiple mechanisms can result in the feedback inhibition of DCs. Foxp3^+^ Tregs bind the co-stimulatory molecules CD80/CD86 on DCs via CTLA-4. This interaction leads to the production of indolamine-2,3-dioxygenase (IDO) and in the suppression of DC maturation (not depicted). IDO causes the apoptosis of conventional T cells and activates Foxp3^+^ Tregs. TGF-β produced and activated by DCs can directly inhibit DC functions. Additionally, signaling via TLRs (e.g., TLR2), DC-SIGN and the Wnt/β-catenin pathway can result in the production of IL-10 by DCs. IL-10 potently inhibits DC maturation/function and induces IL-10-producing T_*R*_1 cells, thereby creating a regulatory feedback loop.

An alternative model is the preferential induction of tolerance by specialized DC subsets (Del Rio et al., 2010; Lutz et al., 2010; Matta et al., 2010; Scott et al., 2011). Here, a division of labor between DC subsets might regulate tolerance versus immunity (Figure 1C). Tolerogenicity may be acquired either inherently or by environmental conditioning of certain DC subsets. However, if certain DC subsets are specialized at inducing tolerance in the steady state, it is still not clear why the *in vivo* elimination of these DC subsets so far failed to elicit autoimmunity (Kaplan et al., 2005; Hildner et al., 2008; Swiecki et al., 2010; Takagi et al., 2011). Therefore, although these different models help to describe certain findings, they fail to fully describe DC-mediated tolerance. Instead, tolerance might be better understood as various interacting and differentially regulated layers that will be dissected in the following paragraphs and in Figure 2.

## Layers of DC-mediated tolerance

### DCs and central tolerance

The thymus produces T cells with random specificities. This creates the problem that potentially harmful autoreactive T cells are readily generated. For this reason, the thymic elimination of such autoreactive T cells (so called negative selection) represents an essential component of tolerance (Figure 2A). Negative selection is thought to involve DCs (Kyewski et al., 1986; Brocker et al., 1997; Volkmann et al., 1997). The majority of thymic DCs localize to the medulla or cortico-medullary junction and comprise CD8α/CD103^+^ cDCs (~50% of total DCs), pDCs (~35%) and CD11b^+^ cDCs (~15%) (Wu and Shortman, 2005). Consistent with DC localization, the thymic medulla is the primary site for negative selection. However, negative selection can also occur at a very early developmental stage in the cortex, with involvement of both cortical thymic epithelial cells (cTECs) and rare cortical DCs (McCaughtry et al., 2008). Most CD8α/CD103^+^cDCs seem to derive locally from thymic T cell precursors, whereas pDCs and CD11b^+^ cDCs likely originate from the circulation, thereby importing peripheral antigens for negative selection (Wu and Shortman, 2005; Bonasio et al., 2006; Li et al., 2009; Hadeiba et al., 2012).

Additionally, non-hematopoietic medullary epithelial cells (mTECs) express and directly present tissue-specific antigens under the control of AIRE (Anderson et al., 2002; Kyewski and Klein, 2006). On the one hand, it was elegantly demonstrated via independent approaches that this DC-independent type of antigen presentation can be sufficient to mediate the negative selection of T cells specific for particular model antigens (Luckashenak et al., 2008; Hinterberger et al., 2010). On the other hand, this may largely depend on the type, localization and concentration of the respective antigen. Indeed, mTEC-mediated negative selection was proposed to be incomplete and to require hematopoietic antigen presenting cells such as medullary DCs which cross-present mTEC-derived antigens (Barclay and Mayrhofer, 1981; Gallegos and Bevan, 2004; Kurobe et al., 2006). This may explain the thymic predominance of the CD8α/CD103^+^ cDC subset which is highly efficient at cross-presentation (Dresch et al., 2011). Thus, DCs likely contribute to central tolerance [(Klein et al., 2011); Figure 2A], although the role of individual DC subsets remain to be established.

### DCs and peripheral tolerance

Several mechanisms of peripheral tolerance operate after T cells have completed their thymic development and have entered the periphery. One is dominant, cell-extrinsic and exerted by Foxp3^+^ Tregs (Figure 2B). In contrast, so-called recessive peripheral tolerance is characterized by a T cell-intrinsic mode of action (Sakaguchi et al., 2008). Examples for recessive tolerance are clonal deletion that results from activation-induced cell death, and clonal anergy which represents a state of functional unresponsiveness (Figure 2C). Finally, negative feedback regulation contributes to peripheral tolerance and will also be considered (Figure 2D).

#### DCs and Foxp3^+^ tregs

Interactions between Foxp3^+^ Tregs and DCs comprise the maintenance/activation of Foxp3^+^ Tregs and their *de novo* differentiation (Figure 2B). In this section, both aspects will be pointed out. Additionally, putative differential regulation upon inflammation and the involvement of DC subsets are discussed.

***Foxp3^+^ Treg maintenance/activation.*** DCs frequently form contacts with Foxp3^+^ Tregs which *in vitro* depend on both antigen and LFA-1 (Tarbell et al., 2004; Sakaguchi et al., 2009). Thereby, Foxp3^+^ Tregs compete with conventional T cells for forming interactions with DCs (Figure 2B). Consequently, the clustering of Foxp3^+^ Tregs around DCs physically inhibits the activation of conventional T cells. Furthermore, Foxp3^+^ Tregs become activated in response to much lower antigen concentrations than conventional T cells even if immature DCs present the antigens (Takahashi et al., 1998; Tarbell et al., 2004; Sakaguchi et al., 2008). Once activated, Foxp3^+^ Tregs proliferate (Figure 2B), enrich at sites of specific antigen encounter, become highly suppressive and may infiltrate inflamed tissues in order to locally confer transient or long-term immune regulation (Yamazaki et al., 2003; Samy et al., 2005; Lahl et al., 2009; Wheeler et al., 2009; Rosenblum et al., 2011). Accordingly, the loss of MHC-class-II expression on DCs results in decreased Treg proliferation and markedly reduced numbers of Foxp3^+^ Tregs (Darrasse-Jeze et al., 2009). Besides antigenic stimulation, Foxp3^+^ Treg homeostasis involves CD28-derived co-stimulatory signals (Figure 2B). In non-obese diabetic (NOD) mice, CD28- or CD80/CD86 deficiency causes drastically reduced numbers of Tregs which results in the onset of autoimmune diabetes (Salomon et al., 2000). Additionally, IL-2- and TGF-β-induced signals were shown to maintain Foxp3 expression, Treg suppressive function, and/or survival [(Marie et al., 2005; Setoguchi et al., 2005; Sakaguchi et al., 2008); Figure 2B]. Treg homeostasis could involve IL-2 trans-presentation by CD25-expressing DCs [(Wuest et al., 2011); Figure 2B]. Moreover, DCs (especially CD8α/CD103^+^ cDCs and intestinal CD103^+^CD11b^+^ cDCs) are potent producers of bioactive TGF-β [(Annacker et al., 2005; Coombes et al., 2007; Travis et al., 2007; Yamazaki et al., 2008; Paidassi et al., 2011); Figure 2B]. Thus, DCs guide the homeostasis of Foxp3^+^ Tregs via multiple mechanisms.

The importance of DCs for the homeostasis of Foxp3^+^ Tregs can be further exemplified by conditions which alter the numbers of DCs *in vivo*. DC-deficient ΔDC mice, CD11c: DTA mice and DT-treated CD11c-DTR bone marrow chimeras all harbor decreased numbers of Foxp3^+^CD25^+^ Tregs with reduced Foxp3 expression (Darrasse-Jeze et al., 2009; Ohnmacht et al., 2009; Bar-on et al., 2011a). This extends to humans with DC deficiency syndromes that are accompanied by reduced numbers of Tregs (reviewed in Collin et al., 2011). Vice versa, an expansion of the mouse DC compartment with exogenous FLT3L induces Treg proliferation (Darrasse-Jeze et al., 2009; Swee et al., 2009).

Are particular DC subsets specialized for the maintenance of Tregs? Given that all DC subsets express significant amounts of MHC-class-II in the immature steady-state, Treg activation seems to be an inherent function of DCs. However, certain DC subsets may more effectively provide additional factors such as IL-2 or active TGF-β. Interestingly, pDCs have been recently shown to control the homeostasis of intestinal Foxp3^+^ Tregs (Takagi et al., 2011). The underlying mechanisms remain to be explored. Foxp3^+^ Treg homeostasis is likely enforced by inflammation which e.g., causes the increased expression of MHC-class-II, CD80, CD86, and IL-2 (Oldenhove et al., 2003; Yamazaki et al., 2003; Banerjee et al., 2006; O'Sullivan et al., 2011). In summary, DCs critically regulate the homeostasis and activation state of Foxp3^+^ Tregs.

***De novo induction of Foxp3^+^ Tregs.*** A second layer of DC/Treg interaction is based on the finding that conventional T cell activation by particular DC subsets can lead to the *de novo* generation of Foxp3^+^ Tregs. This specialization is presumably acquired by DC conditioning and depends on the ability of DCs to produce and/or activate latent TGF-β [(Chen et al., 2003; Paidassi et al., 2011); Figure 2B]. Moreover, DC-derived retinoic acid (RA) enhances the TGF-β-mediated generation of Foxp3^+^ Tregs [(Hill et al., 2008; Mucida et al., 2009); Figure 2B]. RA production in DCs is regulated by the expression of the retinaldehyde-converting enzymes Raldh1 or Raldh2 (Manicassamy and Pulendran, 2009). GM-CSF, RA and TLR signaling (especially via TLR2) represent putative Raldh2-inducing factors within tissues (Manicassamy et al., 2009; Yokota et al., 2009; Guilliams et al., 2010a; Jaensson-Gyllenback et al., 2011). Moreover, β-catenin-dependent signals have been shown to induce TGF-β, Raldh1 and Raldh2 in intestinal DCs (Manicassamy et al., 2010). Additionally, the uptake of apoptotic DCs may imprint Foxp3^+^ Treg-inducing capacity (Kushwah et al., 2009, 2010). As another example, PD-1 ligation on activated T cells was implied in DC-mediated Treg induction [(Fukaya et al., 2010); Figure 2B]. The contribution of these individual mechanisms for Treg induction and the overall role of induced Foxp3^+^ Tregs for tolerance are still under investigation. A recent study suggests that induced Foxp3^+^ Tregs make an important contribution to self tolerance (Haribhai et al., 2011). Moreover, Rudensky and colleagues reported a role for iTregs in suppressing Th2 inflammation at mucosal surfaces (Josefowicz et al., 2012).

Which DC subsets are capable of inducing Foxp3^+^ Tregs? So far, only mesenteric lymph node CD8α/CD103^+^ and CD103^+^CD11b^+^ cDCs, splenic CD8α/CD103^+^ cDCs and subcutaneous lymph node CD11b^+^ cDCs were described to possess endogenous Foxp3^+^ Treg inducing activity (Coombes et al., 2007; Sun et al., 2007; Yamazaki et al., 2008; Guilliams et al., 2010a; Azukizawa et al., 2011). Lung CD8α/CD103^+^ and CD11b^+^ cDC subsets display Raldh2 activity, but have not been functionally tested yet (Guilliams et al., 2010a). In line with these findings, migratory lamina propria CD103^+^CD11b^+^ cDCs were shown to mediate oral tolerance and imprint gut homing properties to T cells including induced Foxp3^+^ Tregs (Jaensson et al., 2008; Schulz et al., 2009; Cassani et al., 2011; Hadis et al., 2011). Furthermore, antigen delivery to CD8α/CD103^+^ cDCs via DEC-205 or DNGR-1 can induce Foxp3^+^ Tregs in the absence of inflammation, yet provokes immunity after co-administration of adjuvants (Kretschmer et al., 2005; Yamazaki et al., 2008; Joffre et al., 2010). This functional dichotomy of CD8α/CD103^+^ cDCs is consistent with the reported abrogation of Treg-inducing functions upon inflammation (Laffont et al., 2010; Hackl et al., 2011). However, DC stimulation by pathogens (e.g., via TLR2) may augment the production of RA and thereby Treg induction (Manicassamy et al., 2009). Indeed, certain infections are known to be accompanied by an expansion of Foxp3^+^ Tregs (Belkaid, 2007; Berod et al., 2012). However, it is still controversial whether this involves genuine *de novo* induction or expansion of pre-existing Foxp3^+^ Tregs. Once induced, Foxp3^+^ Tregs were shown to expand heavily even during immunogenic conditions (Kretschmer et al., 2005). This may reflect the previously discussed increased homeostasis of Foxp3^+^ Tregs by matured *vs*. steady-state DCs (see “Foxp3+ Treg maintenance/activation”). Nevertheless, the suppressive activity of Foxp3^+^ Tregs can be negatively regulated (e.g., by certain inflammatory cytokines), possibly to prevent excessive tolerance (Anz et al., 2010).

One can ask the question why only few DC subsets are endowed with Foxp3^+^ Treg-inducing capacity. This may relate to anatomical localization. For example, mucosal surfaces like the intestine are heavily challenged with microbes. This may lead to the constant triggering e.g., of TLR2 and β-catenin-dependent pathways in intestinal compared to splenic DCs (Manicassamy et al., 2009, 2010). Alternatively, DC subsets could be differentially sensitive toward imprinting signals. Moreover, the stability of imprinting could differ between DC subsets. All of these factors may crystallize toward subset specialization within a dedicated organ (e.g., tolerogenic CD103^+^CD11b^+^ cDCs *versus* immunogenic CD11b^+^ Mo-DCs in the steady state intestine), yet may at the same time explain variability between tissues (e.g., intestine *versus* skin). Markers that distinguish natural from induced Foxp3^+^ Tregs would greatly help to clarify the importance of individual DC subsets for Treg induction. According to recent studies, the previously reported candidate Helios seems to be less specific for natural Tregs than originally thought (Thornton et al., 2010; Akimova et al., 2011; Gottschalk et al., 2012).

#### Recessive peripheral tolerance

Recessive tolerance is a T cell intrinsic process. In the absence of infection, trauma or necrosis, DCs maintain a functionally immature state and continuously process and present self antigens (Reis E Sousa, 2006). Although such recognition of self antigen by T cells can lead to their expansion, they may remain functionally impaired due to clonal anergy (Figure 2C). This process partially depends on CTLA-4 and PD-1 expression by activated T cells [(Nishimura et al., 1999; Freeman et al., 2000; Greenwald et al., 2001; Keir et al., 2006; Fife et al., 2009); Figure 2C and not depicted]. Antigen presentation by DCs can also result in activation-induced cell death (Kurts et al., 1997; Adler et al., 1998; Sakaguchi et al., 2008; Mueller, 2010). This clonal deletion involves Fas-, Bim- or TNF-dependent apoptosis in responding T cells [(Hughes et al., 2008; Hutcheson et al., 2008; Weant et al., 2008; Luckey et al., 2011); Figure 2C]. Apoptotic immune cells or tissue cells were shown to actively enforce this process, partly through suppression of NFκB signaling in DCs (Liu et al., 2002; Rothlin et al., 2007). Notably, steady state DCs can be sufficient to induce immunity in particular settings (Mayerova et al., 2004). It has been discussed that the decision between peripheral tolerance and immunity may be based on only subtle differences that determine how many T cells survive an initial wave of clonal expansion (Reis E Sousa, 2006). The nature of these signals is still a matter of intensive investigation.

Skin, lung, and splenic, if not all CD8α/CD103^+^ DCs are believed to critically maintain CD8^+^ T cell tolerance by cross-presenting cell-associated antigens (Del Rio et al., 2007; Qiu et al., 2009; Henri et al., 2010; Desch et al., 2011). Indeed, in the absence of co-stimulation, antigen targeting to steady state CD8α/CD103^+^ DCs via an antigen-coupled αCD103 antibody was recently shown to promote CD8^+^ T cell tolerance and protection from allergic airway inflammation (Semmrich et al., 2012). Similarly, mice harboring cross-presentation deficient DCs accumulate autoreactive CD8^+^ T cells (Luckashenak et al., 2008). However, clonal deletion can turn into potent immunity upon DC maturation (Hawiger et al., 2001; Bonifaz et al., 2002; Semmrich et al., 2012). Similarly, co-stimulation of CD28 via CD80/CD86 antagonises clonal anergy (Harding et al., 1992). Thus, these mechanisms maintain peripheral tolerance in the steady state, but seem reversible upon inflammation. This pattern of regulation seems opposite to the homeostasis of Foxp3^+^ Tregs.

#### Negative feedback regulation

DCs can also enforce tolerance by initiating negative regulatory feedback loops. Foxp3^+^ Tregs represent one prominent example (Figure 2D). Once activated by DCs, Foxp3^+^ Tregs suppress the production of FLT3L by still incompletely characterized cell populations, resulting in the strictly regulated development of new DCs from their precursors (Kim et al., 2007; Liu et al., 2009; Schildknecht et al., 2010). In the steady state, the cellular sources for FLT3L were recently described to be widespread, including hematopoietic and non-hematopoietic cells, yet their relative contribution to DC development can vary between different tissues (Miloud et al., 2012). In addition to restricting DC numbers, Foxp3^+^ Tregs negatively regulate DC function. This can be achieved by simply outcompeting naïve T cells for interaction (Figure 2B). Furthermore, the binding of CTLA-4 to CD80/CD86 leads to the suppression of DC maturation [(Onishi et al., 2008); Figure 2D]. Accordingly, the relief from Treg-mediated suppression can cause the maturation of DCs (Schildknecht et al., 2010). By suppressing DC maturation, Foxp3^+^ Tregs in turn promote recessive peripheral tolerance (see “Recessive peripheral tolerance”). Another consequence of the CTLA-4-CD80/86 interaction is the release of indolamine-2,3-dioxygenase (IDO) which converts tryptophan into toxic metabolites (Figure 2D). These induce the apoptosis of conventional T cells (Grohmann et al., 2002). Additionally, IDO enforces the suppressive function of Foxp3^+^ Tregs (Sharma et al., 2009). Notably, the disruption of CTLA-4 signals can abrogate the suppressive function of Tregs *in vivo* (Sakaguchi et al., 2008; Wing et al., 2008). This underlines the importance of CTLA-4-mediated feedback inhibition for the suppressive activity of Foxp3^+^ Tregs. Foxp3^+^ Tregs can also secrete TGF-β which results in the *de novo* generation of Foxp3^+^ Tregs and in the suppression of DC functions (Figures 2B and D). TGF-β can be additionally provided by activated T cells or by DCs themselves (Coombes et al., 2007; Rubtsov and Rudensky, 2007; Travis et al., 2007).

The important question remains whether Foxp3^+^ Tregs preferentially engage such regulatory loops in particular DC subsets. Given that FLT3L is a universal growth factor for most DCs and their progenitors, Foxp3^+^ Tregs probably globally control DC homeostasis (Merad et al., 2008; Kingston et al., 2009). Similarly, the targets of CTLA-4 (CD80/CD86) are expressed by most DC subsets. However, it cannot be excluded that Tregs are more potent inhibitors of particular DC subsets by still undefined mechanisms. Interestingly, Treg-dependent DC inhibition does not affect MHC-class-II expression, possibly in order to preserve Foxp3^+^ Treg homeostasis as discussed above (Onishi et al., 2008; Darrasse-Jeze et al., 2009).

IL-10 represents another example for feedback regulation. This cytokine inhibits DC maturation and function. Additionally, IL-10 induces IL-10-producing Foxp3^−^ T_R_1 cells (Figure 2D), thus amplifying negative regulation by so-called infectious tolerance (O'Garra and Vieira, 2007; Barnes and Powrie, 2009). DCs produce IL-10 upon activation e.g., through DC-SIGN-, TLR2- or Wnt/β-catenin-dependent signaling pathways [(Dillon et al., 2006; Manicassamy et al., 2010; Hajishengallis and Lambris, 2011); Figure 2D]. This may especially protect tissues from immune pathology that are continuously challenged with microbial ligands triggering these receptors. This is supported by IL-10-deficient mice which spontaneously suffer from severe colitis (Kuhn et al., 1993). In conclusion, DCs make use of several mechanisms to induce negative regulatory loops which reinforce tolerance. While some of these mechanisms might prevail in the steady state, other loops may primarily serve to counter-regulate inflammation at specific anatomical locations.

### Antigen presentation

DCs acquire exogenous antigens by diverse mechanisms. These include the ingestion of particles (phagocytosis), the engulfment of extracellular fluid (macropinocytosis), and receptor-mediated endocytosis. Exogenous antigens typically gain access to the MHC-class-II presentation pathway. However, these can also be shuttled to the MHC-class-I pathway, a process referred to as cross-presentation (reviewed in Kurts et al., 2010). In contrast, endogenous antigens are classically presented on MHC-class-I molecules.

Since most of the so far dissected mechanisms of tolerance involve antigen recognition, the mode of antigen presentation to T cells also needs to be considered as a component of tolerance. Major insights into such details have been gained by the possibility to target antigens to particular DC subsets *in situ* via the use of antibody conjugates (reviewed in Caminschi and Shortman, 2012). For example, antigen targeting to splenic CD11b^+^ cDCs (via 33D1) or to CD8α/CD103^+^ cDCs (via DEC205) revealed DC-intrinsic specializations for distinct MHC presentation pathways. CD11b^+^ cDCs efficiently form MHC-class-II: peptide complexes whereas CD8α/CD103^+^ cDCs potently cross-present peptides on MHC-class-I molecules (Dudziak et al., 2007). In contrast, steady-state pDCs express low levels of MHC-class-II (Wu and Shortman, 2005). Consequently, CD11b^+^ cDCs may be more efficient at activating existing CD4^+^Foxp3^+^ Tregs compared to CD8α/CD103^+^ cDCs and pDCs, especially when the antigen is limited. In contrast, CD8α/CD103^+^ cDCs efficiently generate active TGF-β, and suboptimal antigen recognition enhances the *de novo* generation of Foxp3^+^ Tregs (Yamazaki et al., 2008). Thus, differences in the antigen uptake capacity and/or expression of the antigen presentation machinery can result in specializations for certain tolerance mechanisms.

The type of uptake receptor triggered on a particular DC can further modulate the resulting immune response. This can be exemplified by the human C-type lectin DC-specific intercellular adhesion molecule-3 grabbing non-integrin (DC-SIGN). This endocytic receptor capable of signaling via Raf-1 is expressed predominantly on human cDCs, yet lacks functionally related homologues in mice. DC-SIGN binds both self antigens and various pathogens including mycobacteria. Mice expressing human DC-SIGN on CD11c^+^ DCs were shown to have reduced immunopathology during mycobacterial infection when compared to wildtype mice (Schaefer et al., 2008). Accordingly, human DC-SIGN^+^ DCs from these infected mice produced significantly less IL-12p40 than wildtype DCs. Additionally, the targeting of DC-SIGN via an antigen-coupled antibody enhances cross-presentation about 1000-fold when compared to unspecific antigen uptake (Tacken et al., 2011). These examples illustrate that the mode of antigen uptake can strongly influence the resulting immune response.

Another important factor is the kinetics of antigen presentation. It has been suggested that persistent antigen presentation may rescue T cells from deletion after their initial priming (Reis E Sousa, 2006). Indeed, antigen targeting to DNGR-1 was shown to induce potent CD4^+^ T cell-dependent antibody responses as a result of persistent antigen presentation (Caminschi and Shortman, 2012). In contrast, poor antibody responses are seen when targeting DEC205 or Clec12A, although all three receptors are expressed by CD8α/CD103^+^ cDCs (Lahoud et al., 2011). Other parameters that can regulate the kinetics of antigen presentation may include the half-life of MHC: peptide complexes on the DC surface, the half-life of DCs themselves and the migration of peripheral DCs into secondary lymphoid organs. Notably, inefficient antigen presentation can result in ignorance as a passive type of tolerance (Mueller, 2010). Thus, the mode, efficiency and kinetics of antigen presentation are important components of tolerance.

## DCs and autoimmunity

**Figure 3:**
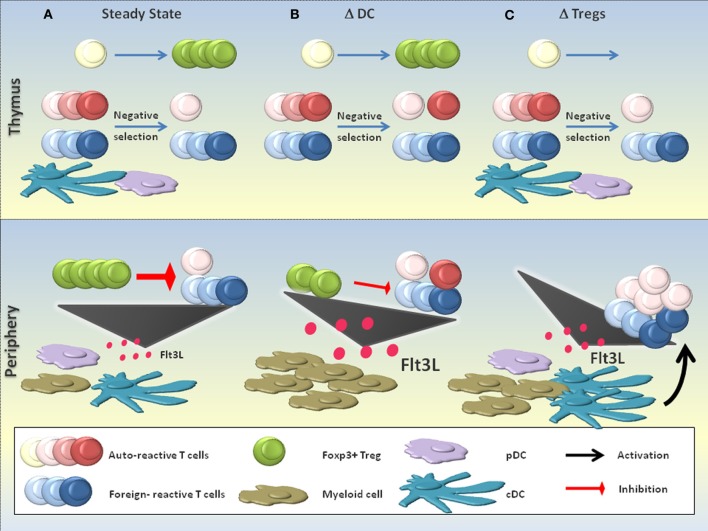
**DCs in the suppression of autoimmunity.** The role of DCs in the suppression of autoimmunity is complex and a matter of discussion. **(A)** In the steady state, DCs are believed to contribute to the negative selection of autoreactive T cells. Few autoreactive T cell clones that escape negative selection can be efficiently controlled by Foxp3^+^ Tregs in the periphery, a process that critically depends on the continuous activation and homeostasis of Tregs by DCs. Equilibrium between the production of the myeloid growth factor FLT3L and its consumption maintains a constant number of DCs and other myeloid cells. **(B)** DC-deficient (ΔDC) mice may exhibit defective negative selection, resulting in the increased seeding of the periphery with self-reactive T cells. Additionally, the absence of peripheral DCs impairs Foxp3^+^ Treg homeostasis and activation, resulting in a substantial reduction of Treg numbers and probably also functionality. The massive accumulation of FLT3L due to the absence of DCs as key consumers of this growth factor leads to a myeloproliferative syndrome. Signs of increased autoreactivity are detectable in ΔDC mice, yet the absence of DCs most likely prevents the activation of self-reactive T cells and thus the full precipitation of systemic autoimmunity. **(C)** The absence of functional Foxp3^+^ Tregs (ΔTregs; e.g., in scurfy mice) is compatible with a normal negative selection, yet provokes severe systemic autoimmunity due to the defective negative regulation of DCs and autoreactive T cells. An increased level of FLT3L in the absence of Foxp3^+^ Tregs expands the numbers of DCs and other myeloid cells.

We have depicted how DCs control multiple layers of tolerance (section “Layers of DC-mediated tolerance”). Consequently, it could be expected that the disruption of DC-mediated tolerance layers or the elimination of DCs that execute such programmes lead to the breakdown of self tolerance. This complex issue can only be briefly discussed (also from a technical point of view) in this section and is summarized in Figure 3.

### Disruption of tolerance layers

Foxp3^+^ Tregs are an important component of DC-dependent tolerance (Figure 2). Indeed, loss-of-function mutations of Foxp3 in mice and men, or the ablation of Foxp3^+^ cells in transgenic mice, all lead to lethal autoimmunity (Brunkow et al., 2001; Gambineri et al., 2003; Kim et al., 2007; Lahl et al., 2007; Sakaguchi et al., 2008). Due to the disrupted negative feedback loop between Foxp3^+^ Tregs and DCs, FLT3L accumulates and DCs expand in Foxp3^+^ Treg-deficient mice [(Liu et al., 2009); Figure 3C]. Additionally, DCs mature (see “Negative feedback regulation”) and prime autoreactive T cells (Figure 3C). Notably, these include tumor-specific T cells (Klages et al., 2010). Moreover, Foxp3^+^ Tregs increase the threshold for foreign-specific immunity which may occur both at the priming and effector phase (Baru et al., 2010; Haque et al., 2010; Suttner et al., 2010; Arnold et al., 2011; Blankenhaus et al., 2011; Dietze et al., 2011; Hadis et al., 2011; Navarro et al., 2011).

TGF-β is another key molecule in tolerance. This cytokine has important immunoregulatory functions, e.g., the maintenance of Foxp3^+^ Tregs and the suppression of DC functions (Figure 2). A failure of DCs to activate latent TGF-β through the α_v_β_8_ integrin results in autoimmunity and colitis (Travis et al., 2007). Moreover, the disruption of TGF-β signaling specifically in DCs renders mice susceptible to experimental autoimmune encephalomyelitis (Laouar et al., 2008). Similarly, the DC-intrinsic disruption of other global inhibitory proteins (e.g., Blimp-1, A20) or the artificial engagement of stimulatory pathways (e.g., Tim-1, CD70) can precipitate immune dysregulation (Keller et al., 2008; Kim et al., 2011; Kool et al., 2011; Xiao et al., 2011). Thus, interfering with DC-mediated tolerance mechanisms can clearly induce autoimmunity (Figure 3C).

### DC ablation

Various mouse models with constitutive DC deficiency are available. Mice deficient in certain transcription factors involved in DC differentiation (like RelB, E2-2 or Batf3) lack specific DC populations, whereas other mutant mice lack several DC subsets (Belz and Nutt, 2012). For example, mice that lack FLT3L, an essential growth factor for DC progenitors, cDCs and pDCs, have severely reduced numbers of DCs (Kingston et al., 2009). Although these models have helped to unveil several aspects of DC development, they often have the main drawback that the transcription factors or growth factors deleted are not DC specific and might therefore affect other immune cell populations. In the last years, two mouse models have been developed in which the suicide gene diphtheria toxin A is expressed in CD11c^+^ cells (ΔDC mice, CD11c: DTA mice). This leads to the constitutive ablation of DCs (Birnberg et al., 2008; Ohnmacht et al., 2009). Moreover, CD11c-DTR mice allow for the inducible depletion of DCs by administration of diphtheria toxin (Jung et al., 2002). By using these mouse models, a role for DCs in the maintenance of tolerance has been suggested. Indeed, increased proportions of Th1 and Th17 cells, hypergammaglobulinemia and/or autoantibody formation have been independently detected in ΔDC-, CD11c:DTA-, FLT3L^−/−^- and inducible DC-depleted mice (Birnberg et al., 2008; Darrasse-Jeze et al., 2009; Ohnmacht et al., 2009). In support of these findings, ΔDC mice exhibit defective negative selection [(Ohnmacht et al., 2009); Figure 3B]. Furthermore, humans with DC, monocyte, B and NK lymphoid (DCML) deficiency can develop autoimmunity (Collin et al., 2011). Altogether, these studies suggest that DC deficiency can result in increased autoreactivity (Figure 3B). However, both ΔDC and CD11c:DTA mice develop a myeloproliferative syndrome that has been explained by the accumulation of FTL3L due to the absence of cDCs (Figure 3B). For this reason, the observed abnormalities of adaptive immune cells have been attributed to the myeloproliferative disease rather than to a primary autoimmune disorder (Birnberg et al., 2008; Bar-on et al., 2011a).

The connection between myeloproliferative disease (as a consequence of DC ablation) and autoimmunity is an intriguing question. FLT3L^−/−^ mice resistant to FLT3L-dependent myeloproliferative disease display features of increased autoreactivity similar to ΔDC and CD11c:DTA mice (Darrasse-Jeze et al., 2009). This extends to humans with DCML deficiency who can develop autoimmune manifestations in the absence of myeloproliferation (Collin et al., 2011). Based on these data, one can argue that increased autoreactivity is not necessarily a consequence of myeloproliferation. Furthermore, the transfer of CD4^+^CD25^+^ Tregs can prevent autoreactivity in DT-treated CD11c-DTR chimeras (Darrasse-Jeze et al., 2009). Foxp3^+^ Treg dysfunction may even contribute to myeloproliferation given that Foxp3^+^ Tregs suppress FLT3L production (Liu et al., 2009). Bar-On et al. use the lack of autoreactivity in CD80/86^−/−^:CD11c:DTA mixed bone marrow chimeras as a supportive argument (Bar-on et al., 2011a). However, one could envisage that CD80/CD86-deficient DCs may sufficiently provide MHC-class-II-dependent signals to Foxp3^+^ Tregs and stimulate their suppressive functions (Sakaguchi et al., 2008; Darrasse-Jeze et al., 2009). Thus, CD80/86^−/−^ DCs may inhibit autoreactivity when compared to DC-deficient settings in which Foxp3^+^ Tregs experience virtually no antigenic stimulation. This does not necessarily conflict with reduced Foxp3^+^ Treg numbers in both scenarios.

More recently, mouse models that allow the depletion of specific DC subsets have been generated to examine the role of particular DC subsets in tolerance and immunity. Langerin (CD207)-positive LCs are located in the epidermis and are believed to take up and transport self antigens to the skin-draining lymph nodes (Waithman et al., 2007). Both inducible (Langerin-DTR) and constitutive (Langerin-DTA) LC depletion models have been developed. However, no obvious onset of skin-specific or systemic autoimmunity has been reported in these LC-ablated mice (Bennett et al., 2005; Kaplan et al., 2005; Kissenpfennig et al., 2005). This is also not the case when the skin of LC-deficient mice is infected with Leishmania major, suggesting that Langerhans cells do not play a major role in suppressing autoimmunity (Kautz-Neu et al., 2011).

Similarly, the contribution of CD8α/CD103^+^ DCs to tolerance has been examined. Although CD103 is not a universal marker for Foxp3^+^ Treg-inducing DCs (Azukizawa et al., 2011), CD8α/CD103^+^ and CD103^+^CD11b^+^ cDCs have prominent steady-state regulatory functions across tissues (Bonifaz et al., 2002; Coombes et al., 2007; Henri et al., 2010). Yet, Batf3^−/−^ mice which constitutively lack CD8α/CD103^+^ DCs, have a normal steady state phenotype (Edelson et al., 2010). Autoimmunity is also not induced upon infections in these mice. In fact, the absence of CD8α/CD103^+^ cDCs results in defective immunity e.g., against influenza, poxvirus, salmonella and West Nile Virus (Hildner et al., 2008; Bogunovic et al., 2009; Beauchamp et al., 2010; Ho et al., 2011). In this case it is thus likely that other DC subsets can compensate for the loss of CD8α/CD103^+^ cDCs to maintain tolerance. Additionally, mice that lack intestinal CD103^+^CD11b^+^ cDCs do not spontaneously develop colitis or autoimmunity. However, severe colitis is induced in these mice upon challenge with dextrane sodium sulphate (Varol et al., 2009). Conversely, an increase in the proportion of CD103^+^CD11b^+^ cDCs by FLT3L treatment results in an increase of Foxp3^+^ Tregs and amelioration of TNF-driven ileitis (Collins et al., 2011). Thus, CD103^+^CD11b^+^ cDCs may balance intestinal immunity in specific situations, but seem to lack a unique tolerogenic function.

Finally, pDCs need to be mentioned. Although pDCs are implied in the pathogenesis of autoimmune diseases like systemic lupus erythematosus, they have also been suggested to promote self tolerance via negative selection and Foxp3^+^ Treg homeostasis (Tian et al., 2007; Matta et al., 2010; Hadeiba et al., 2012). BDCA2-DTR and SiglecH-DTR mice have recently become available to selectively deplete pDCs. Following DT administration, these mice were so far not reported to develop autoreactivity, even after infection with murine cytomegalovirus or vesicular stomatitis virus (Swiecki et al., 2010; Takagi et al., 2011). Whether this is a matter of depletion efficiency and kinetics, or points towards a dispensable role of pDCs in maintaining tolerance, remains to be resolved (also discussed in “Technical aspects of DC ablation”). In conclusion, although there is definite evidence that DCs substantially contribute to prevent autoimmunity by acting at different layers of tolerance (Figure 2; section “Disruption of tolerance layers”), no DC subset has been identified till date which is required to maintain self tolerance in a non-redundant fashion. Constitutive pDC-deficient mice and mice that specifically eliminate CD11b^+^ cDCs remain to be developed in this regard. Moreover, recent studies suggest that certain technical and conceptual aspects need to be considered to resolve these findings.

### Technical aspects of DC ablation

#### Depletion efficiency and side effects

The DC depletion efficiency as well as secondary effects induced by DC depletion can influence the outcome of experiments and their interpretation. One example is represented by ΔDC and CD11c: DTA mice both of which re-activate DTA expression in CD11c^+^ DCs. ΔDC mice lack cDCs, pDCs, and LCs and develop autoimmunity (Ohnmacht et al., 2009). In contrast, CD11c: DTA mice possess pDCs and LCs, but lack cDCs and autoimmunity (Birnberg et al., 2008). This could point toward a critical role of pDCs and LCs in tolerance induction. However, as mentioned, constitutive LC-deficient mice do not develop autoimmunity (Kaplan et al., 2005). Transgenic mice with a constitutive and selective deficiency in pDCs would be very useful to address this still open question. The differential depletion of CD11c^+^ DC subsets in ΔDC and CD11c: DTA mice is likely attributed to different DTA expression levels. Since the same BAC-transgenic CD11c-Cre line was utilized in both cases, these differences could relate to the two different DTA strains used (Birnberg et al., 2008; Ohnmacht et al., 2009).

Furthermore, side effects induced by the ablation of DCs can be problematic. It was recently shown that CD11c-DTR and CD11c-DOG mice develop neutrophilia 6–24 h after DC depletion by injection of DT (Tittel et al., 2012). This is mechanistically distinct from myeloproliferation and is guided by CXCL1/2-dependent recruitment of neutrophils from the bone marrow. According to the timing of DC depletion, this can result in opposing phenotypes in a pyelonephritis model using uropathogenic *Escherichia coli* (Tittel et al., 2012). The reason underlying this early neutrophila is still unclear. One could speculate that the massive apoptosis induced by DT can have side effects on the immune system. Additionally, DT can itself have non-specific toxic effects in wildtype mice, especially when combined with protocols causing activation of the immune system (Meyer Zu Horste et al., 2010). Different DT batches can vary substantially in their toxicity, underlining the requirement for careful titration (Lahl and Sparwasser, 2011). Nevertheless, early neutrophila is not observed when treating wildtype mice with DT or when using CD11c-LuciDTR mice for DC depletion (Tittel et al., 2012). The basis for these findings requires further elucidation.

Additionally, it has been recently shown that DCs interact with high endothelial venules and thereby guide the entry of naïve T cells into lymph nodes (Moussion and Girard, 2011). Consequently, the depletion of DCs may have previously unappreciated side effects on T cell responses by altering the lymph node structure. Whether this relies on particular DC subsets has to be investigated. Taken together, both the depletion efficiency and side effects associated with DC depletion may influence the interpretation of data.

#### Specificity

The promoters and transgene targeting strategies used to drive the ablation of DCs or DC subsets can also strongly influence the outcome of experiments. One classical example is LCs. Three independent models allowing for the inducible or constitutive depletion of LCs based on the C-type lectin Langerin (CD207) generated apparently contradictory results (reviewed in Kaplan et al., 2008). Using Langerin-IRES-DTR-eGFP knock-in mice, no role of LCs for contact hypersensitivity (CHS) reactions was noted (Kissenpfennig et al., 2005). However, Langerin-DTR-eGFP knock-in mice revealed a contribution of LCs to CHS reactions using a similar experimental protocol (Bennett et al., 2005). In contrast, BAC-transgenic Langerin-DTA mice with constitutive LC deficiency develop enhanced CHS responses suggesting a regulatory role of LCs (Kaplan et al., 2005). One important factor contributing to these diverse findings is the specificity of Langerin expression. Langerin is not only expressed by LCs, but also at lower levels by CD8α/CD103^+^ cDCs, as well as on a subset of CD103^−^CD11b^low^ dermal DCs (Kaplan et al., 2008; Henri et al., 2010). Thus, the differential depletion of other Langerin^+^ DC subsets may explain the different outcomes of the CHS reactions. Indeed, only Langerin-DTA mice specifically target LCs. This was explained by the differential regulation of the human *langerin* gene locus that was used to generate Langerin-DTA mice (Kaplan et al., 2008). Therefore, although depletion models have proven to be useful to answer several questions, these models still have limitations and the specificity of cell ablation in a particular model needs to be considered. Of course, also additional factors such as the depletion- and repopulation kinetics, transient *versus* permanent depletion approaches and the microbial environment can have a strong impact on experiments assessing DC functions and autoimmunity. The same applies to both the antigen dose or the administration route used for immunizations. These factors can for example determine whether additional and anatomically distant DC populations have access to antigen.

## Summary and proposed model

In summary, components of the maturation model (Figure 1A), the functional dichotomy model (Figure 1B) and the subset specialization model (Figure 1C) can be combined to describe DC-mediated tolerance. The strict adherence to only one model can explain paradoxical experimental findings like the abrogation of particular tolerance programs during inflammation (outlined in section “Tolerogenic DCs”). At present, we favor the idea that multiple layers of tolerance (Figure 2) are distributed among one or multiple DC subsets. Therefore, any DC can formally be considered “tolerogenic” by executing at least one layer of tolerance. The different layers of tolerance influence each other and can be differentially regulated in order to allow powerful immune responses against pathogens while at the same time restraining autoimmunity.

In the steady state, recessive peripheral tolerance (anergy, deletion) is a default programme of DC-mediated tolerance, given that antigen and antigen-specific T cells are available. On the contrary, only few specialized DCs are capable of *de novo* generation of Foxp3^+^ Tregs, whereas homeostasis and activation of existing Foxp3^+^ Tregs is a global function of DCs. Foxp3^+^ Tregs negatively control both DC numbers and maturation and thereby enforce steady-state tolerance. Inflammatory signals cause functional DC maturation that antagonises recessive peripheral tolerance. Similarly, Treg-inducing properties can be abrogated, whereas the activation and homeostasis of existing Foxp3^+^ Tregs continue or are even enforced. Foxp3^+^ Tregs disrupt DC activity and thereby counter-regulate immunity at different levels. Additional mechanisms of feedback regulation can be promoted, e.g., via production of IL-10. As DCs continue to execute tolerogenic programmes during inflammation, tolerance and immunity may represent parallel events tentatively executed by the same DC. Yet certain subsets can be specialized in particular layers of tolerance and some layers may be more resistant toward inflammatory signals than others. This, in the sum, allows the initiation of immunity, whereas at the same time self tolerance is actively maintained.

Past research using DC depletion models has provided important insights into DC-mediated tolerance, yet the ablation of DC subsets so far failed to induce autoimmunity. If one excludes technical aspects as an explanation, one could postulate that a non-redundant tolerogenic DC subset simply doesn't exist. Moreover, it should be considered that DCs are required to induce (auto)immunity. Thus, the global breakdown of steady-state tolerance can be largely masked in the absence of DCs (Figure 3). This would explain why fatal autoimmunity occurs when disrupting non-redundant layers of tolerance (such as Foxp3^+^ Tregs) while DCs remain present (Figure 3C). Unraveling the complex mechanisms of DC- and Foxp3^+^ Treg-mediated immune regulation across all layers might be the key to the selective therapeutic manipulation of the immune system. This could allow for the enhancement of immunity against pathogens and tumors while suppressing unwanted self-specific responses, and *vice versa*.

### Conflict of interest statement

The authors declare that the research was conducted in the absence of any commercial or financial relationships that could be construed as a potential conflict of interest.
